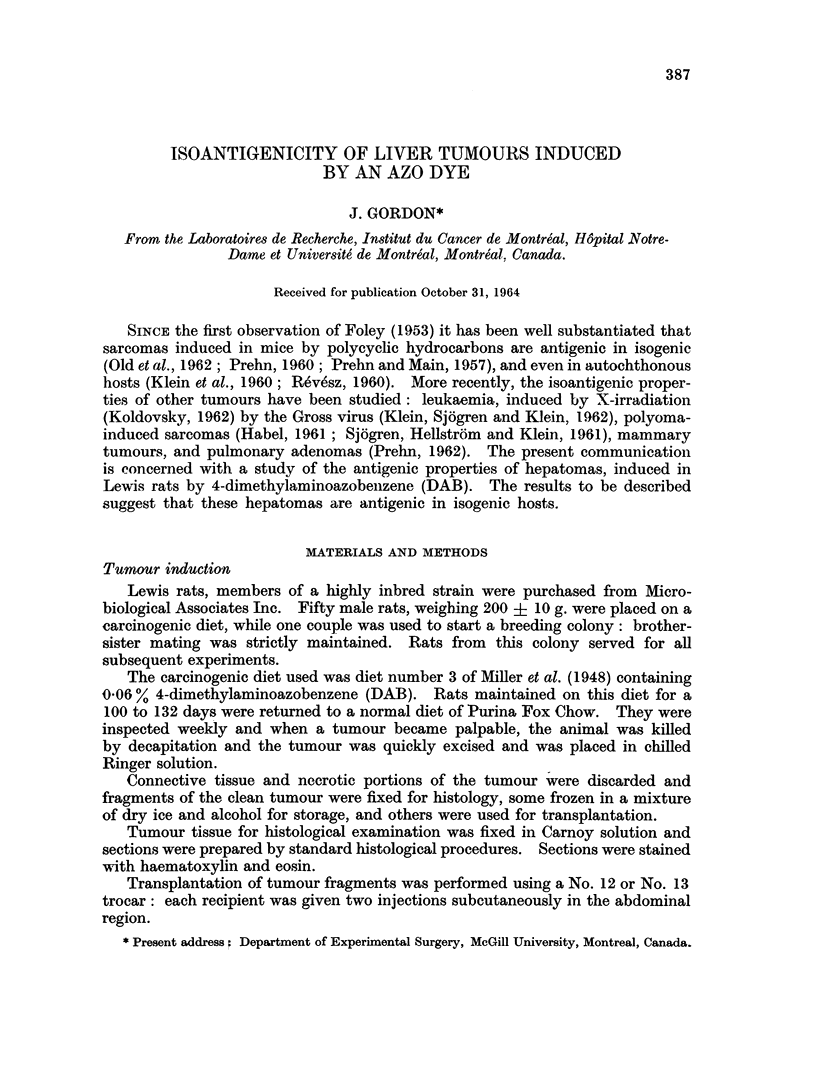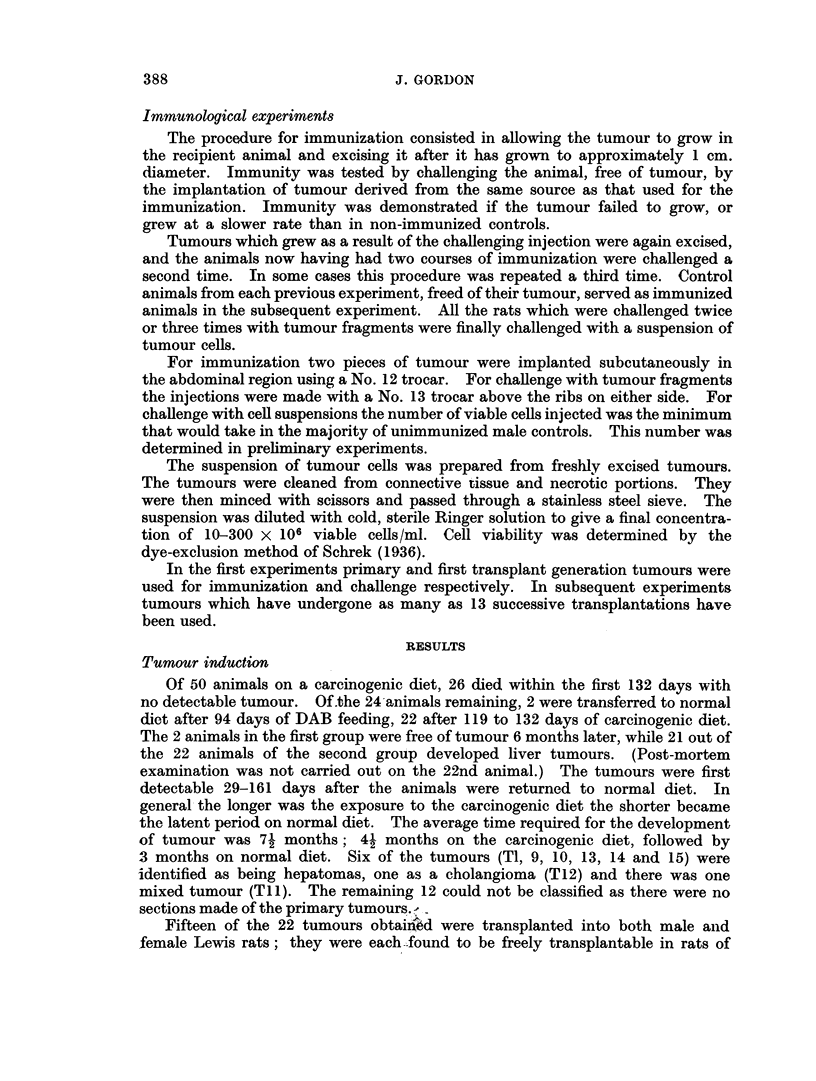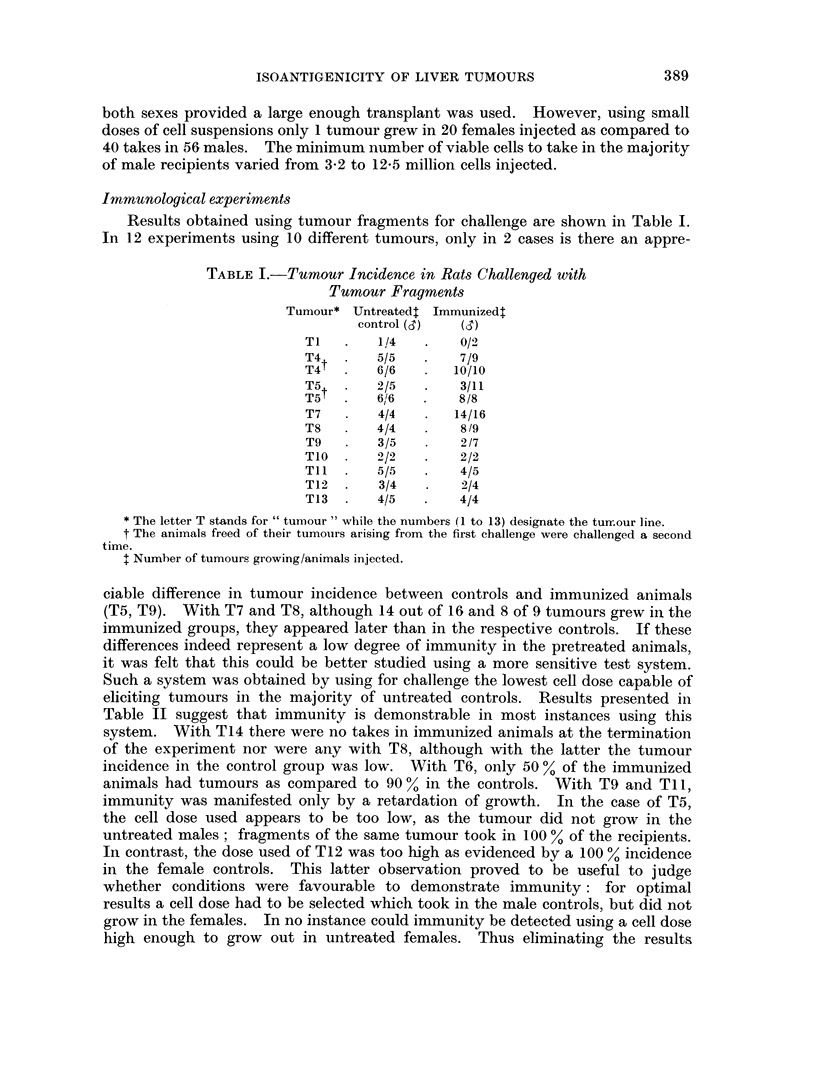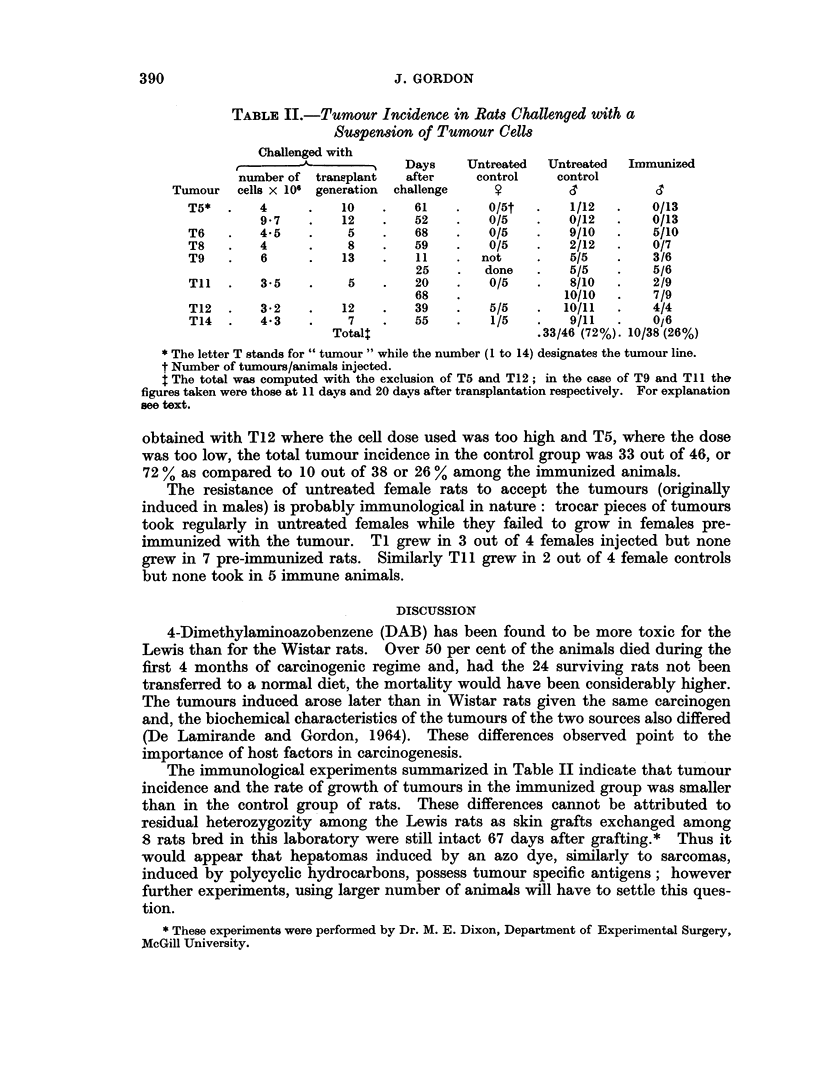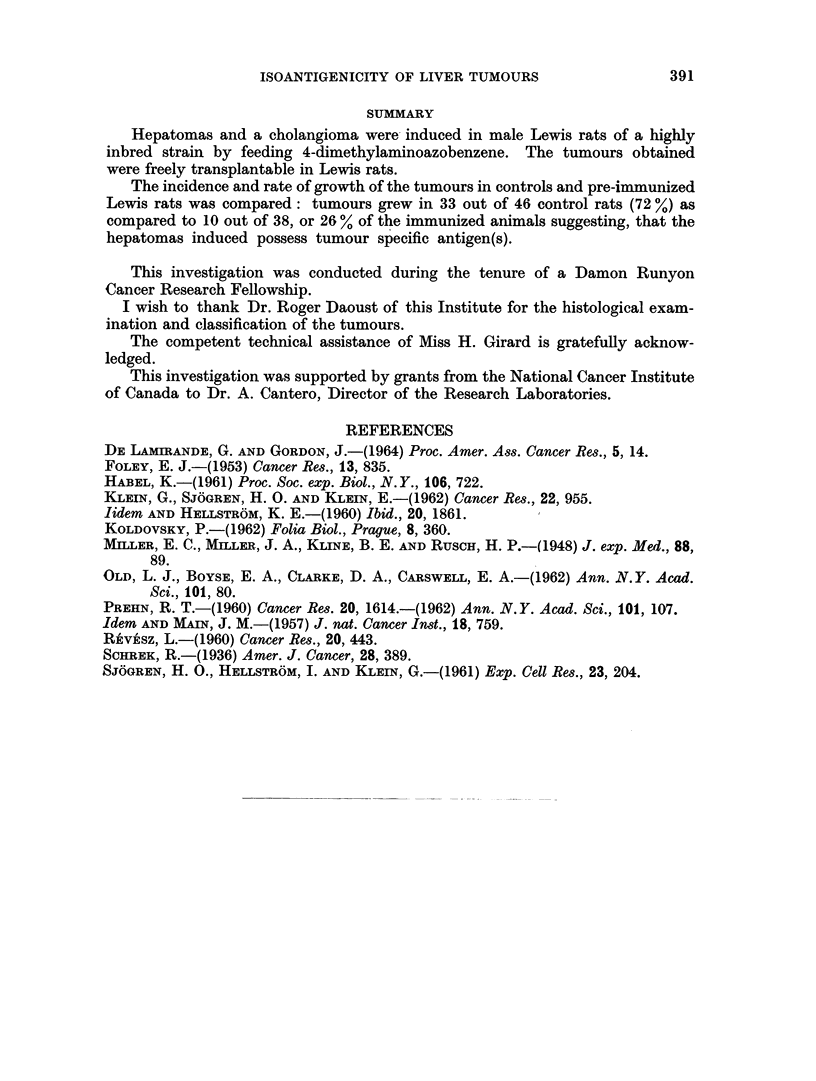# Isoantigenicity of Liver Tumours Induced by an Azo Dye

**DOI:** 10.1038/bjc.1965.46

**Published:** 1965-06

**Authors:** J. Gordon


### ISOANTIGENICITY OF LIVER TUMOURS INDUCED BY AN AZO DYE

J. GORDON*

*From the Laboratoires de Recherche, Institut du Cancer de Montréal, Hôpital Notre-Dame et Université de Montréal, Montréal, Canada.*

Received for publication October 31, 1964

SINCE the first observation of Foley (1953) it has been well substantiated that sarcomas induced in mice by polycyclic hydrocarbons are antigenic in isogenic (Old *et al.*, 1962 ; Prehn, 1960 ; Prehn and Main, 1957), and even in autochthonous hosts (Klein *et al.*, 1960 ; Révész, 1960). More recently, the isoantigenic properties of other tumours have been studied : leukaemia, induced by X-irradiation (Koldovsky, 1962) by the Gross virus (Klein, Sjögren and Klein, 1962), polyoma-induced sarcomas (Habel, 1961 ; Sjögren, Hellström and Klein, 1961), mammary tumours, and pulmonary adenomas (Prehn, 1962). The present communication is concerned with a study of the antigenic properties of hepatomas, induced in Lewis rats by 4-dimethylaminoazobenzene (DAB). The results to be described suggest that these hepatomas are antigenic in isogenic hosts.

### MATERIALS AND METHODS

### *Tumour induction*

Lewis rats, members of a highly inbred strain were purchased from Microbiological Associates Inc. Fifty male rats, weighing 200 $\pm$ 10 g. were placed on a carcinogenic diet, while one couple was used to start a breeding colony : brother-sister mating was strictly maintained. Rats from this colony served for all subsequent experiments.

The carcinogenic diet used was diet number 3 of Miller *et al.* (1948) containing 0·06 % 4-dimethylaminoazobenzene (DAB). Rats maintained on this diet for a 100 to 132 days were returned to a normal diet of Purina Fox Chow. They were inspected weekly and when a tumour became palpable, the animal was killed by decapitation and the tumour was quickly excised and was placed in chilled Ringer solution.

Connective tissue and necrotic portions of the tumour were discarded and fragments of the clean tumour were fixed for histology, some frozen in a mixture of dry ice and alcohol for storage, and others were used for transplantation.

Tumour tissue for histological examination was fixed in Carnoy solution and sections were prepared by standard histological procedures. Sections were stained with haematoxylin and eosin.

Transplantation of tumour fragments was performed using a No. 12 or No. 13 trocar : each recipient was given two injections subcutaneously in the abdominal region.

* Present address : Department of Experimental Surgery, McGill University, Montreal, Canada.

*Immunological experiments*

The procedure for immunization consisted in allowing the tumour to grow in the recipient animal and excising it after it has grown to approximately 1 cm. diameter. Immunity was tested by challenging the animal, free of tumour, by the implantation of tumour derived from the same source as that used for the immunization. Immunity was demonstrated if the tumour failed to grow, or grew at a slower rate than in non-immunized controls.

Tumours which grew as a result of the challenging injection were again excised, and the animals now having had two courses of immunization were challenged a second time. In some cases this procedure was repeated a third time. Control animals from each previous experiment, freed of their tumour, served as immunized animals in the subsequent experiment. All the rats which were challenged twice or three times with tumour fragments were finally challenged with a suspension of tumour cells.

For immunization two pieces of tumour were implanted subcutaneously in the abdominal region using a No. 12 trocar. For challenge with tumour fragments the injections were made with a No. 13 trocar above the ribs on either side. For challenge with cell suspensions the number of viable cells injected was the minimum that would take in the majority of unimmunized male controls. This number was determined in preliminary experiments.

The suspension of tumour cells was prepared from freshly excised tumours. The tumours were cleaned from connective tissue and necrotic portions. They were then minced with scissors and passed through a stainless steel sieve. The suspension was diluted with cold, sterile Ringer solution to give a final concentration of 10–300 $\times$ $10^6$ viable cells/ml. Cell viability was determined by the dye-exclusion method of Schrek (1936).

In the first experiments primary and first transplant generation tumours were used for immunization and challenge respectively. In subsequent experiments tumours which have undergone as many as 13 successive transplantations have been used.

### RESULTS

*Tumour induction*

Of 50 animals on a carcinogenic diet, 26 died within the first 132 days with no detectable tumour. Of the 24 animals remaining, 2 were transferred to normal diet after 94 days of DAB feeding, 22 after 119 to 132 days of carcinogenic diet. The 2 animals in the first group were free of tumour 6 months later, while 21 out of the 22 animals of the second group developed liver tumours. (Post-mortem examination was not carried out on the 22nd animal.) The tumours were first detectable 29–161 days after the animals were returned to normal diet. In general the longer was the exposure to the carcinogenic diet the shorter became the latent period on normal diet. The average time required for the development of tumour was 7½ months; 4½ months on the carcinogenic diet, followed by 3 months on normal diet. Six of the tumours (Tl, 9, 10, 13, 14 and 15) were identified as being hepatomas, one as a cholangioma (T12) and there was one mixed tumour (T11). The remaining 12 could not be classified as there were no sections made of the primary tumours.

Fifteen of the 22 tumours obtained were transplanted into both male and female Lewis rats; they were each found to be freely transplantable in rats of both sexes provided a large enough transplant was used. However, using small doses of cell suspensions only 1 tumour grew in 20 females injected as compared to 40 takes in 56 males. The minimum number of viable cells to take in the majority of male recipients varied from 3·2 to 12·5 million cells injected.

*Immunological experiments*

Results obtained using tumour fragments for challenge are shown in Table I. In 12 experiments using 10 different tumours, only in 2 cases is there an appre-

TABLE I.—*Tumour Incidence in Rats Challenged with Tumour Fragments*

| Tumour* | Untreated‡ control (♂) | Immunized‡ (♂) |
|---|---|---|
| T1 | 1/4 | 0/2 |
| T4 | 5/5 | 7/9 |
| T4† | 6/6 | 10/10 |
| T5 | 2/5 | 3/11 |
| T5† | 6/6 | 8/8 |
| T7 | 4/4 | 14/16 |
| T8 | 4/4 | 8/9 |
| T9 | 3/5 | 2/7 |
| T10 | 2/2 | 2/2 |
| T11 | 5/5 | 4/5 |
| T12 | 3/4 | 2/4 |
| T13 | 4/5 | 4/4 |

* The letter T stands for " tumour " while the numbers (1 to 13) designate the tumour line.
† The animals freed of their tumours arising from the first challenge were challenged a second time.
‡ Number of tumours growing/animals injected.

ciable difference in tumour incidence between controls and immunized animals (T5, T9). With T7 and T8, although 14 out of 16 and 8 of 9 tumours grew in the immunized groups, they appeared later than in the respective controls. If these differences indeed represent a low degree of immunity in the pretreated animals, it was felt that this could be better studied using a more sensitive test system. Such a system was obtained by using for challenge the lowest cell dose capable of eliciting tumours in the majority of untreated controls. Results presented in Table II suggest that immunity is demonstrable in most instances using this system. With T14 there were no takes in immunized animals at the termination of the experiment nor were any with T8, although with the latter the tumour incidence in the control group was low. With T6, only 50% of the immunized animals had tumours as compared to 90% in the controls. With T9 and T11, immunity was manifested only by a retardation of growth. In the case of T5, the cell dose used appears to be too low, as the tumour did not grow in the untreated males ; fragments of the same tumour took in 100% of the recipients. In contrast, the dose used of T12 was too high as evidenced by a 100% incidence in the female controls. This latter observation proved to be useful to judge whether conditions were favourable to demonstrate immunity : for optimal results a cell dose had to be selected which took in the male controls, but did not grow in the females. In no instance could immunity be detected using a cell dose high enough to grow out in untreated females. Thus eliminating the results

TABLE II.—*Tumour Incidence in Rats Challenged with a Suspension of Tumour Cells*

| Tumour | Challenged with: number of cells × $10^6$ | Challenged with: transplant generation | Days after challenge | Untreated control ♀ | Untreated control ♂ | Immunized ♂ |
|---|---|---|---|---|---|---|
| T5* | 4 | 10 | 61 | 0/5† | 1/12 | 0/13 |
| | 9·7 | 12 | 52 | 0/5 | 0/12 | 0/13 |
| T6 | 4·5 | 5 | 68 | 0/5 | 9/10 | 5/10 |
| T8 | 4 | 8 | 59 | 0/5 | 2/12 | 0/7 |
| T9 | 6 | 13 | 11 | not | 5/5 | 3/6 |
| | | | 25 | done | 5/5 | 5/6 |
| T11 | 3·5 | 5 | 20 | 0/5 | 8/10 | 2/9 |
| | | | 68 | | 10/10 | 7/9 |
| T12 | 3·2 | 12 | 39 | 5/5 | 10/11 | 4/4 |
| T14 | 4·3 | 7 | 55 | 1/5 | 9/11 | 0/6 |
| | | Total‡ | | | 33/46 (72%) | 10/38 (26%) |

* The letter T stands for " tumour " while the number (1 to 14) designates the tumour line.
† Number of tumours/animals injected.
‡ The total was computed with the exclusion of T5 and T12 ; in the case of T9 and T11 the figures taken were those at 11 days and 20 days after transplantation respectively. For explanation see text.

obtained with T12 where the cell dose used was too high and T5, where the dose was too low, the total tumour incidence in the control group was 33 out of 46, or 72 % as compared to 10 out of 38 or 26 % among the immunized animals.

The resistance of untreated female rats to accept the tumours (originally induced in males) is probably immunological in nature : trocar pieces of tumours took regularly in untreated females while they failed to grow in females pre-immunized with the tumour. T1 grew in 3 out of 4 females injected but none grew in 7 pre-immunized rats. Similarly T11 grew in 2 out of 4 female controls but none took in 5 immune animals.

### DISCUSSION

4-Dimethylaminoazobenzene (DAB) has been found to be more toxic for the Lewis than for the Wistar rats. Over 50 per cent of the animals died during the first 4 months of carcinogenic regime and, had the 24 surviving rats not been transferred to a normal diet, the mortality would have been considerably higher. The tumours induced arose later than in Wistar rats given the same carcinogen and, the biochemical characteristics of the tumours of the two sources also differed (De Lamirande and Gordon, 1964). These differences observed point to the importance of host factors in carcinogenesis.

The immunological experiments summarized in Table II indicate that tumour incidence and the rate of growth of tumours in the immunized group was smaller than in the control group of rats. These differences cannot be attributed to residual heterozygozity among the Lewis rats as skin grafts exchanged among 8 rats bred in this laboratory were still intact 67 days after grafting.* Thus it would appear that hepatomas induced by an azo dye, similarly to sarcomas, induced by polycyclic hydrocarbons, possess tumour specific antigens ; however further experiments, using larger number of animals will have to settle this question.

* These experiments were performed by Dr. M. E. Dixon, Department of Experimental Surgery, McGill University.

SUMMARY

Hepatomas and a cholangioma were induced in male Lewis rats of a highly inbred strain by feeding 4-dimethylaminoazobenzene. The tumours obtained were freely transplantable in Lewis rats.

The incidence and rate of growth of the tumours in controls and pre-immunized Lewis rats was compared: tumours grew in 33 out of 46 control rats (72%) as compared to 10 out of 38, or 26% of the immunized animals suggesting, that the hepatomas induced possess tumour specific antigen(s).

This investigation was conducted during the tenure of a Damon Runyon Cancer Research Fellowship.

I wish to thank Dr. Roger Daoust of this Institute for the histological examination and classification of the tumours.

The competent technical assistance of Miss H. Girard is gratefully acknowledged.

This investigation was supported by grants from the National Cancer Institute of Canada to Dr. A. Cantero, Director of the Research Laboratories.

### REFERENCES

De Lamirande, G. and Gordon, J.—(1964) *Proc. Amer. Ass. Cancer Res.*, **5**, 14.
Foley, E. J.—(1953) *Cancer Res.*, **13**, 835.
Habel, K.—(1961) *Proc. Soc. exp. Biol., N.Y.*, **106**, 722.
Klein, G., Sjögren, H. O. and Klein, E.—(1962) *Cancer Res.*, **22**, 955.
*Iidem* and Hellström, K. E.—(1960) *Ibid.*, **20**, 1861.
Koldovsky, P.—(1962) *Folia Biol., Prague*, **8**, 360.
Miller, E. C., Miller, J. A., Kline, B. E. and Rusch, H. P.—(1948) *J. exp. Med.*, **88**, 89.
Old, L. J., Boyse, E. A., Clarke, D. A., Carswell, E. A.—(1962) *Ann. N.Y. Acad. Sci.*, **101**, 80.
Prehn, R. T.—(1960) *Cancer Res.* **20**, 1614.—(1962) *Ann. N.Y. Acad. Sci.*, **101**, 107.
*Idem* and Main, J. M.—(1957) *J. nat. Cancer Inst.*, **18**, 759.
Révész, L.—(1960) *Cancer Res.*, **20**, 443.
Schrek, R.—(1936) *Amer. J. Cancer*, **28**, 389.
Sjögren, H. O., Hellström, I. and Klein, G.—(1961) *Exp. Cell Res.*, **23**, 204.